# Radiotherapy exposure directly damages the uterus and causes pregnancy loss

**DOI:** 10.1172/jci.insight.163704

**Published:** 2023-03-22

**Authors:** Meaghan J. Griffiths, Sarah A. Marshall, Fiona L. Cousins, Lauren R. Alesi, Jordan Higgins, Saranya Giridharan, Urooza C. Sarma, Ellen Menkhorst, Wei Zhou, Alison S. Care, Jacqueline F. Donoghue, Sarah J. Holdsworth-Carson, Peter A.W. Rogers, Evdokia Dimitriadis, Caroline E. Gargett, Sarah A. Robertson, Amy L. Winship, Karla J. Hutt

**Affiliations:** 1 Department of Anatomy and Developmental Biology, Development and Stem Cells Program, Biomedicine Discovery Institute, Monash University, Clayton, Victoria, Australia.; 2 Department of Obstetrics and Gynaecology, University of Melbourne, Parkville, Victoria, Australia.; 3 Gynaecology Research Centre, The Royal Women’s Hospital, Parkville, Victoria, Australia.; 4 Department of Obstetrics and Gynaecology, Monash University, Clayton, Victoria, Australia.; 5 The Ritchie Centre, Hudson Institute of Medical Research, Clayton, Victoria, Australia.; 6 Robinson Research Institute and Adelaide Medical School, University of Adelaide, Adelaide, South Australia, Australia.; 7 Epworth HealthCare, Richmond, Victoria, Australia.

**Keywords:** Reproductive Biology, Vascular Biology, Apoptosis, Clinical practice, Mouse models

## Abstract

Female cancer survivors are significantly more likely to experience infertility than the general population. It is well established that chemotherapy and radiotherapy can damage the ovary and compromise fertility, yet the ability of cancer treatments to induce uterine damage, and the underlying mechanisms, have been understudied. Here, we show that in mice total-body γ-irradiation (TBI) induced extensive DNA damage and apoptosis in uterine cells. We then transferred healthy donor embryos into ovariectomized adolescent female mice that were previously exposed to TBI to study the impacts of radiotherapy on the uterus independent from effects to ovarian endocrine function. Following TBI, embryo attachment and implantation were unaffected, but fetal resorption was evident at midgestation in 100% of dams, suggesting failed placental development. Consistent with this hypothesis, TBI impaired the decidual response in mice and primary human endometrial stromal cells. TBI also caused uterine artery endothelial dysfunction, likely preventing adequate blood vessel remodeling in early pregnancy. Notably, when pro-apoptotic protein Puma-deficient (*Puma^–/–^*) mice were exposed to TBI, apoptosis within the uterus was prevented, and decidualization, vascular function, and pregnancy were restored, identifying PUMA-mediated apoptosis as a key mechanism. Collectively, these data show that TBI damages the uterus and compromises pregnancy success, suggesting that optimal fertility preservation during radiotherapy may require protection of both the ovaries and uterus. In this regard, inhibition of PUMA may represent a potential fertility preservation strategy.

## Graphical abstract

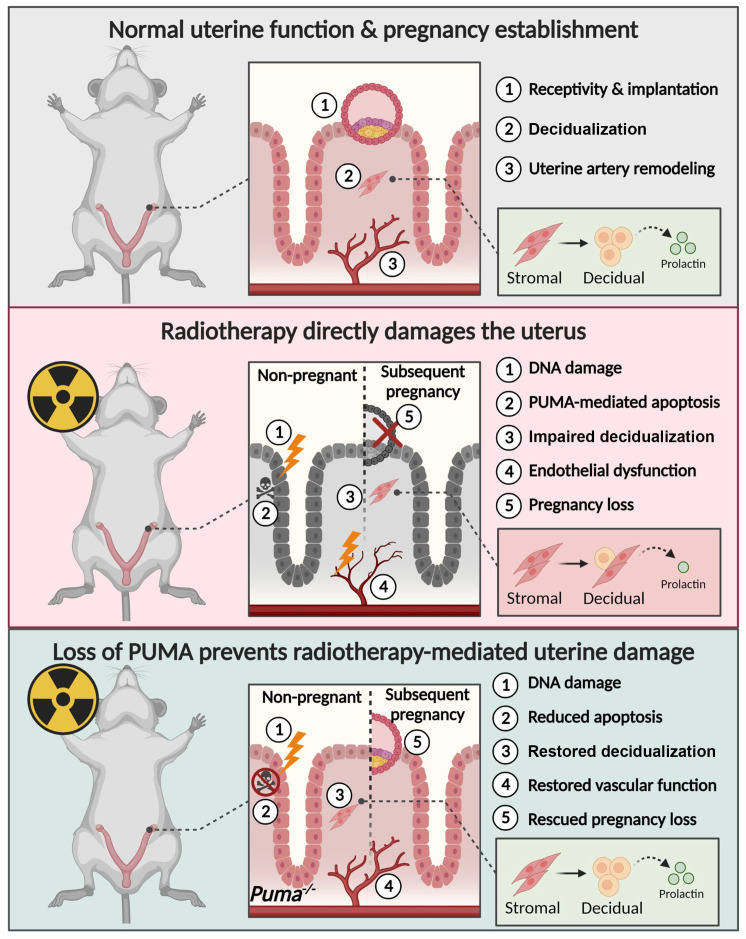

## Introduction

With 5-year survival rates for young cancer patients at an all-time high (1), enhancing the long-term quality of life for survivors has emerged as a new priority. Up to 80% of reproductive-age female cancer survivors (i.e., under 40 years old) will experience diminished fertility after treatment, compared with their healthy counterparts (2–7). It is well established that radiotherapy and certain chemotherapies can cause significant ovarian damage, often leading to infertility and premature menopause (8–13). However, our understanding of how cancer treatments might impact the uterus, and the establishment and maintenance of healthy pregnancy, is incomplete. Additionally, the mechanisms underlying uterine damage and pregnancy complications following cancer treatment are unclear.

Clinical data suggest that the uterus may sustain persistent damage following radiotherapy treatment, particularly in young, prepubescent girls treated with total-body, abdominal, or pelvic irradiation (14–17). Notably, however, prepubescent girls exposed to irradiation have smaller uterine volumes following hormone treatment than age-matched controls (18–21). This outcome could be due to lack of endogenous hormone production (i.e., exogenous hormone support does not fully compensate for endogenous hormone production during puberty), or it could be the result of cancer treatment–induced damage to the prepubescent uterus.

As adults, reduced clinical pregnancy rates (22, 23) and increased rates of pregnancy complications (24–30) and adverse pregnancy outcomes (11, 17, 19, 31–39) are reported in survivors. Critically, these complications are evident in survivors treated both pre- and postpubertally with a treatment regimen consisting of radiotherapy alone or in combination with chemotherapy. While many patients deliver healthy infants (40, 41), early pregnancy loss (42, 43), preterm delivery (26, 34, 44), low birth weight (27), small-for-gestational-age infants (25, 34), stillbirth (45), and uterine rupture during delivery (39) all occur at disproportionately higher rates in survivors compared with women without a history of cancer treatment (46). In addition to impacting maternal health, such complications are associated with long-term consequences for the infant, such as impaired cardiovascular and neurological outcomes, which may lead to cerebral palsy or increased risk of developing heart disease, diabetes, and hypertension later in life (47).

Studies investigating pregnancy rates and outcomes in cancer survivors who use assisted reproductive technologies provide additional evidence to indicate the uterus may be directly damaged following cancer treatment. For example, even when oocyte or embryo cryopreservation are undertaken prior to cancer treatment, or donor oocytes or embryos are utilized, survivors are less likely to achieve pregnancy from the first embryo transfer cycle (42). Moreover, survivors with comparable ovarian hormone profiles and numbers of retrieved oocytes to control patients require more embryo transfers to achieve pregnancy (31). In other words, even when healthy, unexposed oocytes or embryos are used, or adequate ovarian hormone function is present, pregnancy success remains impaired in these women. Together, these observations indicate that uterine exposure to radiotherapy may cause lasting damage to the uterus. However, it is unclear if impaired uterine function occurs indirectly as a result of diminished endocrine support of uterine receptivity for pregnancy (due to ovarian damage) or if the uterus itself is directly damaged.

Examination of the direct impacts of radiotherapy treatment on uterine function using human patient data is inherently difficult as cancer treatment is often patient specific. Furthermore, treatment regimens rarely consist of single agents, making it difficult to characterize the specific impacts of different cancer treatments on the uterus, subsequent fertility, and pregnancy outcomes. This mechanistic information is a vital step toward the development of effective strategies to preserve uterine function and ensure healthy pregnancy for women surviving cancer. Therefore, to address these knowledge gaps, we employed an in vivo mouse model utilizing clinically relevant regimens of total-body irradiation (TBI), combined with in vitro studies using irradiated primary human endometrial epithelial and stromal cells, to elucidate the impacts of uterine radiotherapy exposure on the establishment and maintenance of healthy pregnancy.

## Results

### Radiotherapy exposure causes direct uterine cell DNA damage and apoptosis.

**Figure 1:**
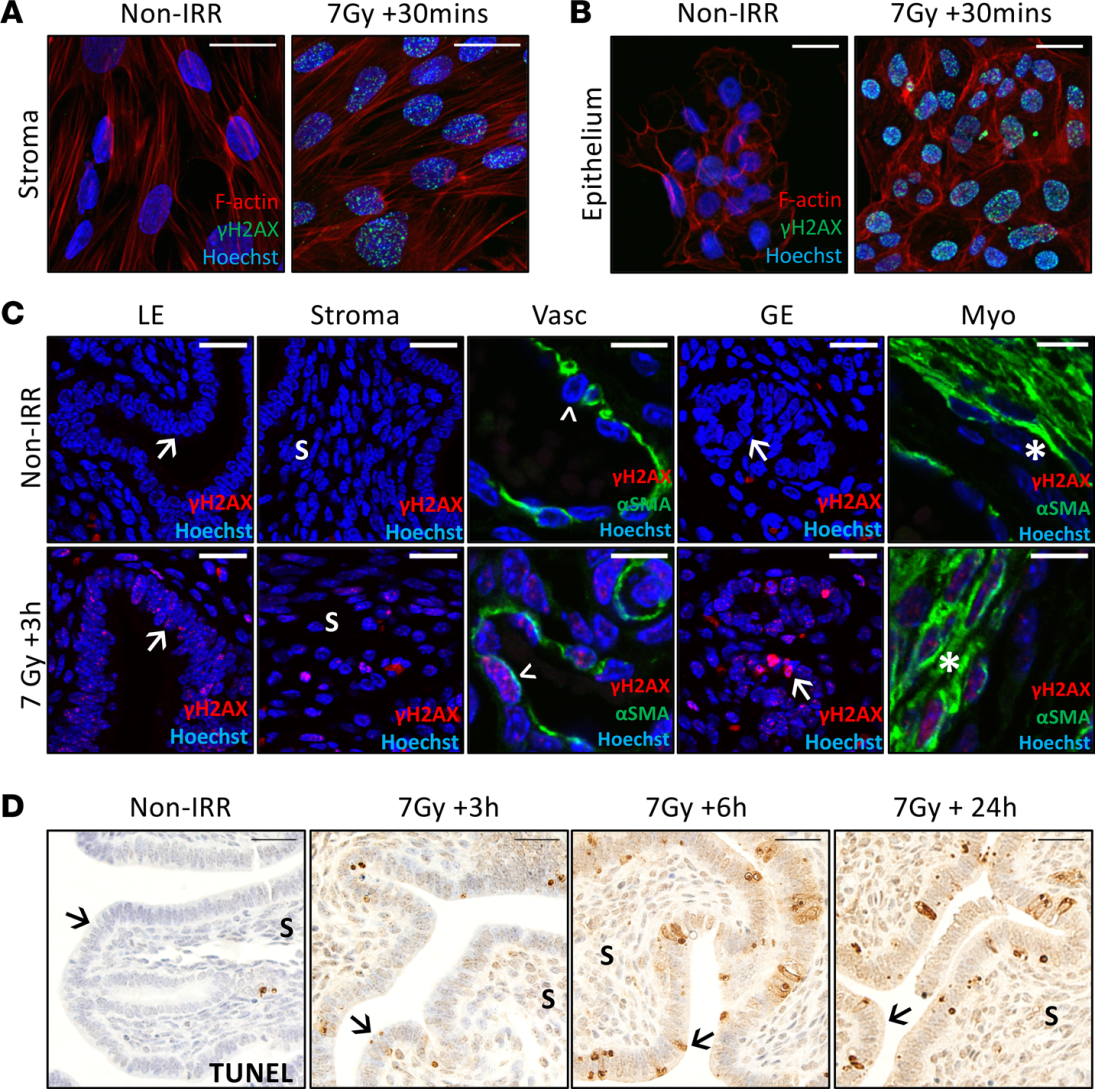
Radiotherapy exposure induces direct uterine DNA damage and apoptosis in vitro and in vivo. (**A**) Primary endometrial stromal cells and (**B**) immortalized human endometrial epithelial cells were exposed to 7 Gy γ-irradiation or left as nonirradiated controls (Non-IRR) (*n* = 3 passages, in duplicate). Representative immunofluorescence images at 30 minutes postirradiation are shown. (**C**) Adolescent female wild-type mice exposed to 7 Gy TBI (IRR) or nonirradiated controls (Non-IRR) were collected after 3 hours (h) to examine the immediate effects of radiotherapy on the uterus via cells positive for γH2AX DNA damage in each cellular compartment (*n* = 4/group). (**D**) Representative images of TUNEL-stained uterine sections at 3, 6, and 24 hours postirradiation are shown. Scale bars are 25 μm;→ luminal epithelium; S stroma; > pericyte; * myometrium; LE, luminal epithelium; GE, glandular epithelium.

First, we aimed to establish if radiotherapy induces direct uterine cell damage in human cells, as this has not been previously demonstrated to our knowledge. Strikingly, irradiation of human endometrial stromal (Figure 1A) and epithelial cells (Figure 1B) resulted in extensive DNA damage within 30 minutes of exposure.

Next, we determined whether the uterus is sensitive to radiotherapy-induced damage, in vivo. We employed pubertal mice (4 weeks old) for this study, as TBI is frequently administered in children and adolescents as a myeloablative conditioning treatment for leukemia prior to stem cell or bone marrow transplantation (48). Since radiation is typically delivered as multiple fractions in these patients (48), mice were exposed to 7 Gy γ-irradiation (delivered in 2 fractions of 3.5 Gy), and uteri were collected 3 hours, 6 hours, or 24 hours later. Consistent with our observations in human cells, as early as 3 hours postirradiation, widespread DNA damage was observed within all major cell compartments of the uterus — including the uterine epithelium, stroma, myometrium, and pericytes lining the blood vessels — as shown by positive γH2AX staining (Figure 1C). TUNEL staining indicates that this DNA damage led to extensive apoptosis from 3–24 hours postirradiation (Figure 1D).

The γ-irradiation regimen was well tolerated in vivo. By 4 weeks postirradiation, local uterine immune cell numbers (with the exception of neutrophils) were unchanged compared to nonirradiated control animals, despite significant depletion of the same immune cell types peripherally in the spleen (Supplemental Figures 1 and 2; supplemental material available online with this article; https://doi.org/10.1172/jci.insight.163704DS1). Since uterine immune cells play fundamental roles in facilitating early pregnancy establishment and maintenance, we considered 4 weeks to be an appropriate recovery time to investigate uterine function postirradiation in vivo, in the context of pregnancy. This also allowed sufficient time to promote cell turnover and the repair of induced DNA damage within the uterus.

### Early pregnancy milestones are unaffected by radiotherapy exposure.

To identify how milestones of pregnancy establishment may be influenced by radiotherapy exposure, hormone priming for endometrial receptivity — a prerequisite for uterine blastocyst implantation — was administered using a well-established protocol (49) (Supplemental Figure 3A). Uterine weight was significantly reduced after hormone treatment in irradiated mice compared with nonirradiated controls, indicating perturbed hormone responsiveness demonstrating a potentially irreversible impact of irradiation exposure on the endometrial tissue of the pubertal uterus (Supplemental Figure 3, B and C). This finding is consistent with reports in young women with a previous history of uterine radiotherapy exposure, who typically have a reduced uterine volume and endometrial thickness, even after hormone replacement therapy (18–21). Despite differences in uterine weight between groups, many molecular hallmarks of endometrial receptivity were induced equally in the uteri of both nonirradiated control and irradiated animals. This was indicated by low levels of the antiadhesive proteins, mucin 1 and E-cadherin protein, localization in the endometrial luminal epithelium, as well as a distinct absence of Ki67^+^ proliferative cells at the apical surface of the endometrium in the luminal epithelium (Supplemental Figure 3D). Furthermore, gene expression levels of key endometrial receptivity mediators were similar in uterine tissues between groups (Supplemental Figure 3E). This suggested a defect in epithelial or stromal cell proliferation or turnover, as opposed to altered differentiation states or transcriptional program in endometrial cells.

**Figure 2:**
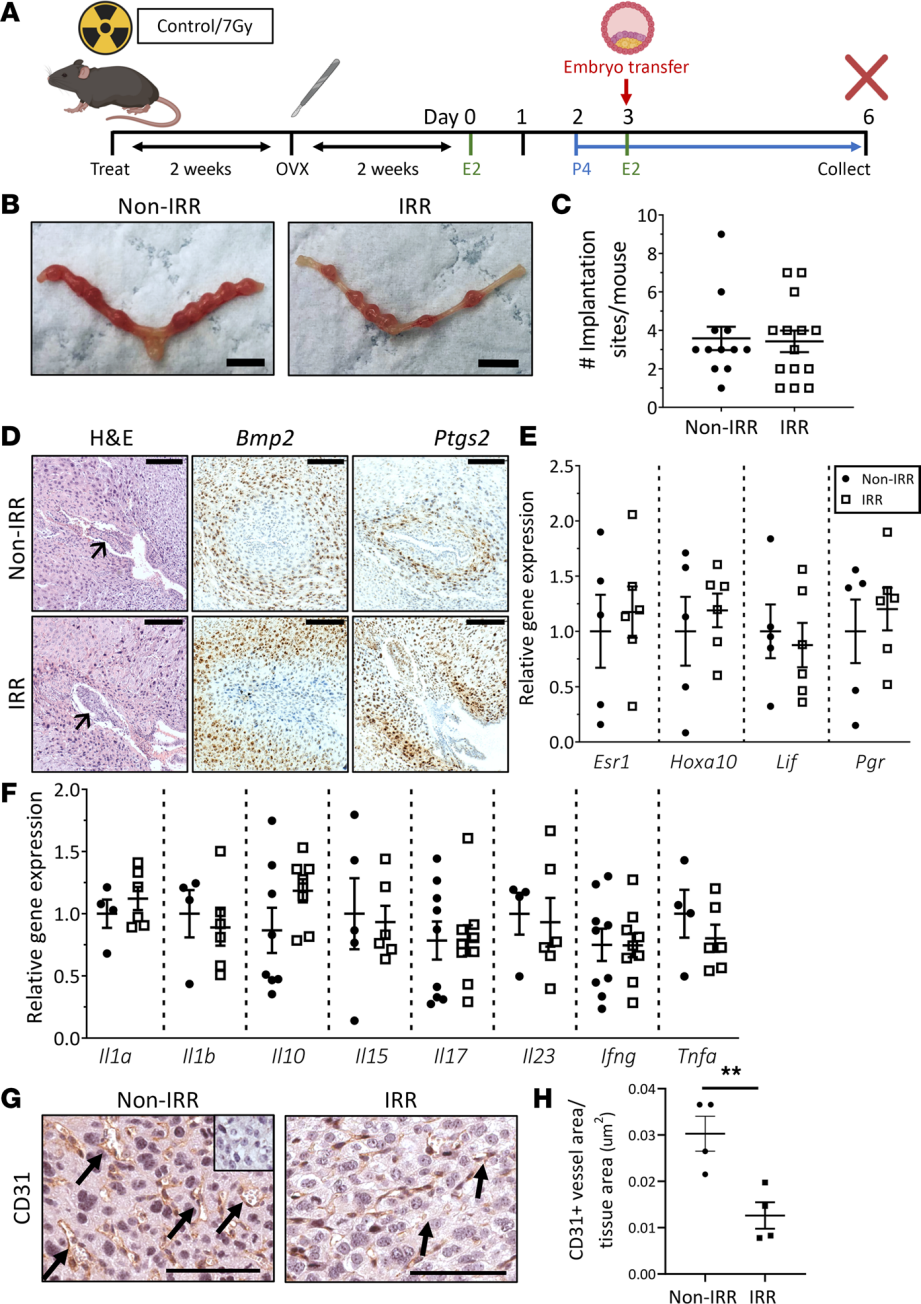
Radiotherapy exposure does not impair endometrial receptivity or early blastocyst implantation. (**A**) Ovariectomized (OVX) adolescent female wild-type mice exposed to 7 Gy TBI (IRR) or nonirradiated controls (Non-IRR) were hormone-primed with estradiol (E2) and progesterone (P4) and received healthy donor embryo transfers. (**B**) Representative images of uteri collected on day 6, 3 days posttransfer, are shown. (**C**) The number of uterine implantation sites per mouse was quantified. (**D**) Representative images of embryo attachment sites and in situ hybridization staining for gene markers of and early implantation are shown. Arrows point to the developing embryo in the implantation site. (**E**) Expression of genes critical to the implantation process — *Esr1*, *Hoxa10*, *Lif*, and *Pgr* — was analyzed by quantitative PCR (qPCR). (**F**) Uterine cytokine gene expression was evaluated in implantation sites by qPCR. (**G**) Implantation sites immunostained for CD31 (negative control inset). Arrows point to CD31-positive blood vessels. (**H**) Total CD31-positive vessel area relative to total tissue section area is significantly reduced following irradiation. Scale bars are 5 mm (**B**), 50 μm (**D**), 100 μm (**G**). Data are mean ± SEM; unpaired *t* test (2 groups; parametric distribution) or Mann-Whitney test (2 groups; nonparametric distribution); *n* = 12–14/group, **P < 0.01.

Subsequently, we designed an animal model to determine the impact of radiotherapy-mediated uterine damage on pregnancy establishment and maintenance. For these studies, mice were either exposed to a single 4.5 Gy dose or cumulative 7 Gy dose of γ-irradiation or left unexposed as control mice. All animals were ovariectomized (i.e., ovaries surgically removed; OVX mice) and supplied with exogenous hormones, prior to the transfer of embryos from healthy, unexposed donors (Figure 2A) as described previously (50, 51). Importantly, this strategy enabled us to precisely investigate the effects of radiotherapy-mediated uterine damage on the maternal uterine contribution to pregnancy, independent from impacts to ovarian endocrine function and/or embryo quality.

At 3 days after embryo transfer, blastocyst attachment rates were comparable between nonirradiated control and irradiated mice (Figure 2, B and C). Implantation site weight was also unchanged (Supplemental Figure 4). Within implantation sites, the gross morphology and developmental progression of the attachment sites were similar between groups (Figure 2D). In support, in situ hybridization localization patterns of key implantation genes, bone morphogenetic protein 2 (*Bmp2*) and prostaglandin-endoperoxide synthase 2 (*Ptgs2*), were similar between groups (Figure 2D). Furthermore, gene expression levels of hormone receptors (estrogen receptor 1 [*Esr1*], progesterone receptor [*Pgr*]), key implantation mediators (homeobox A10 [*Hoxa10*], leukemia inhibitory factor [*Lif*]) (Figure 2E), as well as cytokines (Figure 2F) were unchanged between groups. Quantification of CD31-positive blood vessel area revealed significantly reduced cross-sectional vascular area in the decidua following irradiation (Figure 2, G and H). Together, these data demonstrate that uterine receptivity to early events of blastocyst attachment and trophoblast invasion are not significantly impacted by radiotherapy exposure; however, reduced blood vessel area at this early pregnancy milestone may be critical to ongoing pregnancy success.

### The maternal decidualization response to pregnancy is impaired following radiotherapy.

**Figure 3:**
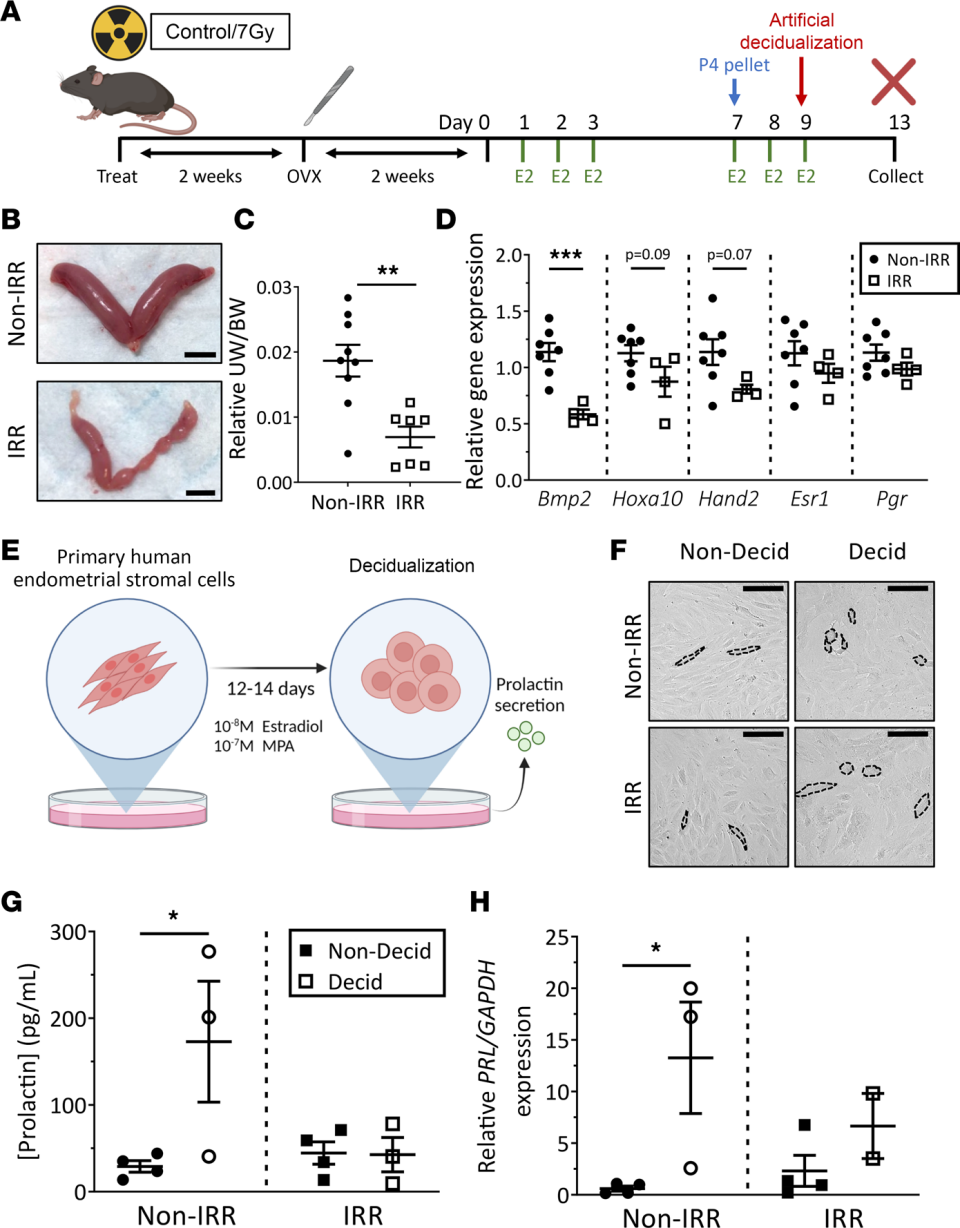
Radiotherapy exposure impairs decidualization in vitro and in vivo. (**A**) Ovariectomized (OVX) adolescent female wild-type mice exposed to 7 Gy TBI (IRR) or nonirradiated controls (Non-IRR) were hormone-primed with estradiol (E2) and a progesterone pellet (P4) before undergoing artificial decidualization. (**B**) Representative images of uteri collected 4 days after artificial decidualization are shown. (**C**) Relative uterine weight (UW) to body weight (BW) was quantified. (**D**) Expression of genes key for decidualization and hormone responses — *Esr1* and *Pgr* — was analyzed by qPCR. (**E**) Cultured primary human endometrial stromal fibroblasts were exposed to 7 Gy γ-irradiation (IRR) or left as nonirradiated controls (Non-IRR) and then artificially decidualized in vitro. (**F**) Representative images highlighting cell morphology between nondecidualized (Non-Decid) and decidualized (Decid) cells are shown. (**G**) The concentration of prolactin in the media was quantified by ELISA. (**H**) Prolactin (PRL) gene expression was assessed by qPCR. Scale bars: 5 mm. Data are mean ± SEM; unpaired *t* test (2 groups; parametric distribution), Mann-Whitney test (2 groups; nonparametric distribution) or 1-way ANOVA with Holm-Šídák post hoc test; *P < 0.05, **P < 0.01, ***P < 0.001; *n* = 2–9/group. Hand2, heart and neural crest derivatives expressed 2.

We next explored the impact of radiotherapy on endometrial stromal cell decidualization, a hormone-driven event that is essential for ongoing pregnancy and early placental development (52). Decidualization involves terminal differentiation of endometrial stromal cells into large, round decidual cells. Decidualization was artificially induced in mice as previously described (53) to study the impact of irradiation on maternal adaptations to pregnancy, independent of blastocyst influence (Figure 3A). Notably, the decidualization response was markedly reduced in irradiated mice, with areas of irradiated uteri failing to decidualize properly (Figure 3B). Accordingly, a significantly decreased relative uterine weight to body weight was recorded in irradiated mice compared with nonirradiated controls (Figure 3C). Interestingly, at this time point, genes encoding the hormone receptors *Esr1* and *Pgr* were unchanged between treatment groups (Figure 3D). In contrast, transcript levels of the hormonally regulated target gene and key regulator of decidualization — *Bmp2* *—* were significantly reduced in irradiated uteri compared with nonirradiated controls (Figure 3D). Trends in reduced expression of *Hoxa10* and heart and neural crest derivatives expressed 2 (*Hand2*), which are also critical regulators of decidualization and hormone responsiveness, were observed in irradiated uteri yet were not statistically significant (Figure 3D). Together, the in vivo mouse model demonstrates a reduced decidual mass, possibly due to deficient stromal cell decidualization, and suggests reduced hormone responsiveness as a consequence of radiotherapy exposure.

Next, using primary human endometrial stromal cells in vitro, we sought to determine whether γ-irradiation directly acts on the stromal cells to impair their differentiation during decidualization. Primary human endometrial stromal fibroblasts isolated from endometrial tissue biopsies were cultured and exposed to 7 Gy γ-irradiation, then artificially decidualized in vitro as previously described (54, 55) (Figure 3E). Morphologically, some irradiated cells appeared to become rounded and to decidualize similarly to nonirradiated control cells (Figure 3F). However, it was evident that while nonirradiated control stromal fibroblast cells displayed significant increases in prolactin secretion (Figure 3G) and gene expression (Figure 3H), irradiated cells did not exhibit the same increases. Although these data are variable, they provide good preliminary evidence that radiotherapy exerts a direct impact on primary human endometrial stromal fibroblasts that impairs their ability to decidualize.

### Radiotherapy-induced uterine damage causes pregnancy loss in a mouse model.

Having established that the uterus sustains direct damage following radiotherapy, and the maternal decidualization response was impaired, we utilized our embryo transfer animal model to determine the impact of radiotherapy-mediated uterine damage on pregnancy maintenance.

**Figure 4:**
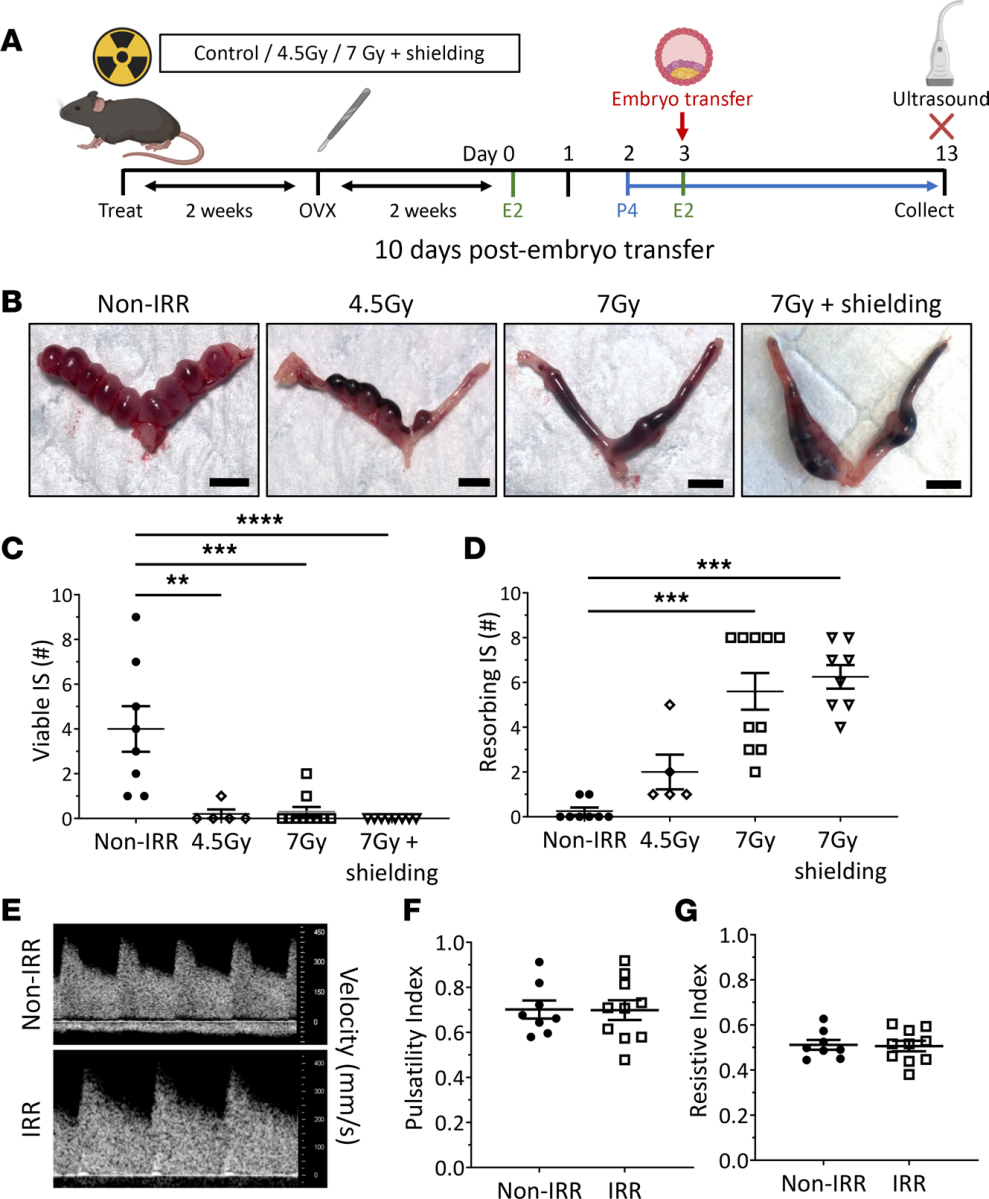
Radiotherapy exposure causes midgestation pregnancy loss. (**A**) Ovariectomized (OVX) adolescent female wild-type mice were exposed to either 4.5 Gy or 7 Gy TBI, exposed to 7 Gy lower body (i.e., + shielding) γ-irradiation, or left as nonirradiated controls (Non-IRR). Mice were hormone-primed with estradiol (E2) and progesterone (P4) and received healthy donor embryo transfers. (**B**) Representative images of uteri collected on day (d) 13, 10 days posttransfer, are shown. The numbers of viable (**C**) and resorbing (**D**) implantation sites were quantified. Uterine artery blood flow was assessed on pregnant mice using Doppler ultrasonography immediately before collection. (**E**) Representative images of uterine artery waveforms are shown. Pulsatility (**F**) and resistive (**G**) indexes were quantified. Scale bars: 5 mm. Data are mean ± SEM; unpaired *t* test (2 groups; parametric distribution) or Kruskal-Wallis test (>2 groups; nonparametric distribution); **P < 0.01, ***P < 0.001, **** *P* < 0.0001; *n* = 5–10/group.

At 10 days after embryo transfer (Figure 4A), implantation sites developed normally in nonirradiated control mice (Figure 4B). However, complete pregnancy loss was observed in the majority of irradiated animals, with a significant reduction in the number of viable implantation sites and increased resorbing implantation sites (Figure 4, B–D). These data suggest that radiotherapy-induced uterine damage, as opposed to altered embryo developmental competence, leads to pregnancy loss. Critically, the observed pregnancy loss did not correlate with measurable differences in uterine artery function. The uterine artery waveform shape (Figure 4E), and uterine artery pulsatility (Figure 4F) and resistance indices (Figure 4G) detected by ultrasonography, were unchanged between groups. These data raise the possibility that uterine damage in human patients may occur after radiotherapy but may be undetectable by noninvasive means.

It is established that TBI can damage the brain and so may potentially disrupt the hypothalamic-pituitary-gonadal (HPG) axis and alter hormone production. To investigate the prospect that irradiation-induced HPG hormone production contributes to pregnancy loss, brain shielding was employed during TBI. Lead shields were positioned to ensure only abdominopelvic exposure to irradiation (Figure 4B). Consistent with the previous results in mice exposed to TBI, significantly fewer viable implantation sites and increased resorbing sites were observed following abdominopelvic irradiation (Figure 4, B–D). This indicates that uterine dysfunction and pregnancy loss following irradiation are not caused by damage to the HPG axis.

### Radiotherapy exposure is associated with uterine artery endothelial dysfunction.

**Figure 5:**
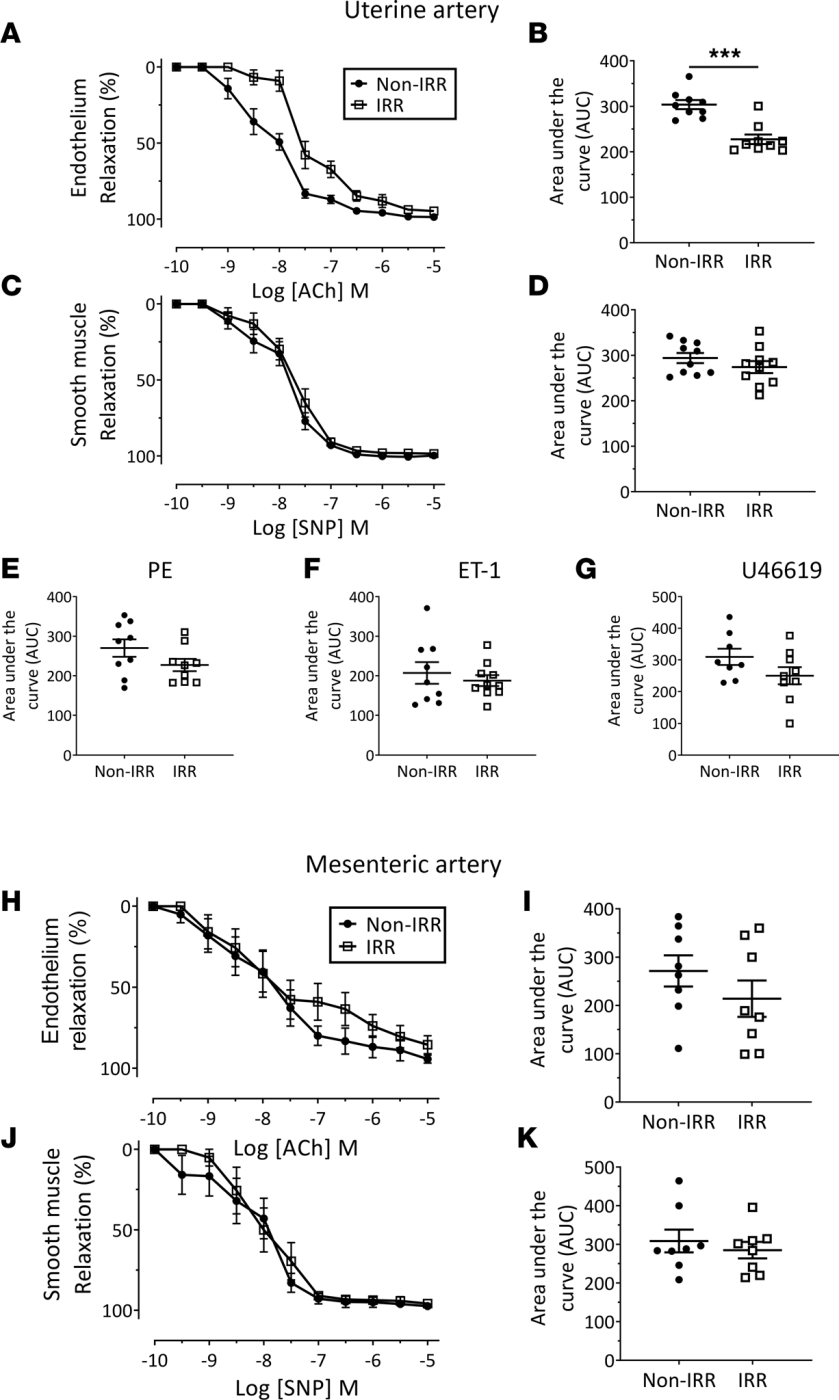
Radiotherapy exposure is associated with uterine artery endothelial dysfunction. Endothelium-dependent (**A** and **B**) and smooth muscle–dependent (**C** and **D**) relaxation were assessed by addition of ACh and SNP, respectively, with concentration versus percentage relaxation and AUC shown. Vasoconstriction was also assessed by addition of PE (**E**), ET-1 (**F**), and U46619 (**G**), and AUC was quantified. Analysis of mesenteric arteries from the same animals was performed simultaneously and showed no differences in endothelium-dependent (**H** and **I**) or smooth muscle–dependent (**J** and **K**) relaxation. Data are mean ± SEM; unpaired *t* test (2 groups; parametric distribution) or Mann-Whitney test (2 groups; nonparametric distribution); ***P < 0.001; *n* = 8–10/group.

Notably, at 3 days after embryo transfer, the uteri of irradiated mice were pale and avascular in appearance, compared with uteri of nonirradiated controls (Figure 4B and Supplemental Figure 3B). Adequate blood flow is critical for development of the placenta and maintenance of a successful pregnancy (56, 57). Clinical data demonstrate that uterine artery pulsatility is increased in nonpregnant patients previously exposed to radiotherapy, suggesting that blood vessel function is impaired postirradiation (17). These data, combined with our observations that uterine vascular cells sustained radiotherapy-induced DNA damage and underwent immediate apoptosis (Figure 1, A and B), and of reduced CD31^+^ vessel density in irradiated implantation sites (Figure 2, G and H), led us to hypothesize that the uterine vasculature may be functionally impaired by irradiation. Indeed, by performing wire myography experiments, we found that endothelium-dependent relaxation of the uterine artery was impaired in mice at 4 weeks postirradiation, with the area under the curve (AUC) significantly reduced (Figure 5, A and B, and Table 1). Smooth muscle–dependent relaxation (Figure 5, C and D, and Table 1) was unchanged in response to irradiation. While the vasoconstrictive ability of the uterine artery demonstrated enhanced sensitivity (pEC_50_) to only PE and U46619, this was not significant when analyzed by AUC (Figure 5, E–G, and Table 1). Notably, irradiation-induced deficits to endothelium-dependent relaxation were unique to the uterine artery, since mesenteric artery function displayed no significant differences between the treatment groups (Figure 5, H–K, and Supplemental Table 1). Thus, radiotherapy-induced damage to the uterine artery was endothelium specific and persisted long term and so was likely to contribute to pregnancy loss following TBI.

### Radiotherapy-induced uterine damage is mediated by PUMA.

**Figure 6:**
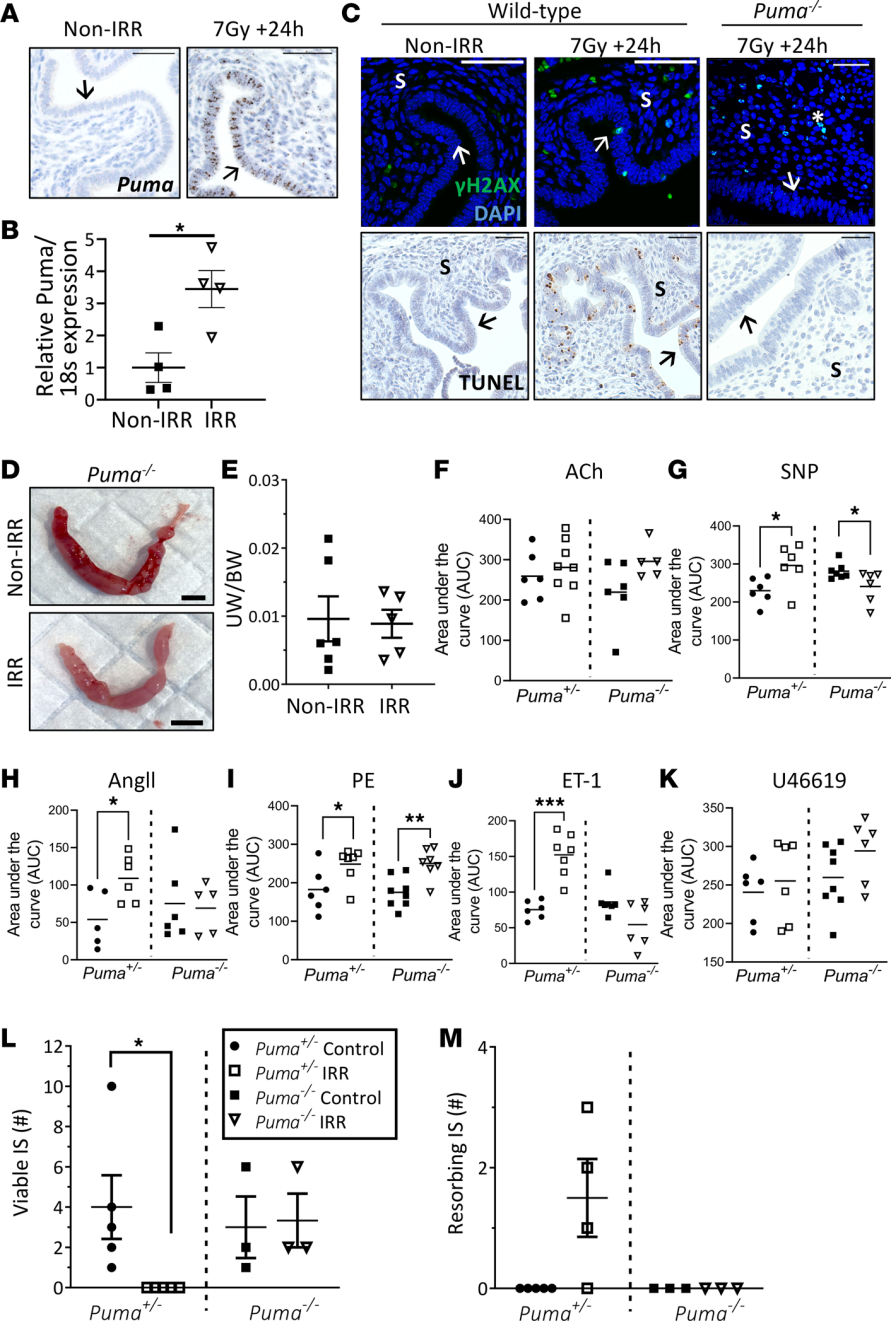
Loss of PUMA protects against radiotherapy-mediated uterine damage. Adolescent female *Puma^+/–^* or *Puma^–/–^* mice were exposed to 7 Gy TBI or left as nonirradiated controls (Non-IRR). (**A**) Uterine Puma mRNA expression was localized by in situ hybridization (bars = 50 μm) and (**B**) quantified by qPCR 24 hours postirradiation. (**C**) Representative images of immunofluorescence and TUNEL-stained uterine sections from wild-type or *Puma^–/–^* mice at 24 hours postirradiation. (**D**) Ovariectomized (OVX) adolescent female *Puma^–/–^* mice exposed to 7 Gy TBI or nonirradiated controls were hormone-primed with estradiol (E2) and a progesterone pellet (P4) before undergoing artificial decidualization. Representative images of uteri collected 4 days after artificial decidualization are shown. (**E**) Uterine weight (UW) to body weight (BW) was quantified. (**F**) Female *Puma^+/–^* or *Puma^–/–^* mice were exposed to 7 Gy TBI or left as nonirradiated controls, then 4 weeks postirradiation had uterine artery function assessed by wire myography. Vessel relaxation (**F** and **G**) and vasoconstriction (**H**–**K**) were assessed by area under the curve (AUC). Ovariectomized adolescent female *Puma^+/–^* or *Puma^–/–^* mice were hormone-primed with estradiol and progesterone and received healthy donor embryo transfers 4 weeks postirradiation. The number of viable (**L**) and resorbing (**M**) implantation sites were quantified. Scale bars are 50 μm (**A** and **C**) and 5 mm (**D**); → luminal epithelium; S stroma; * endothelium. Data are mean ± SEM; unpaired *t* test (2 groups; parametric distribution) or Mann-Whitney test (2 groups; nonparametric distribution), *P < 0.05, **P < 0.01, ***P < 0.001. *n* = 3–8/group.

Knowing that radiotherapy induces extensive DNA damage and apoptosis in both mouse and human uterine cells (Figure 1), we next aimed to define the mechanisms underpinning this damage more precisely. PUMA, a pro-apoptotic BH3-only protein belonging to the intrinsic (i.e., mitochondrial) apoptosis pathway, is responsible for rapidly triggering apoptosis in oocytes following exposure to γ-irradiation (58). PUMA activation in the uterus has not previously been described to our knowledge; therefore, we first aimed to localize and quantify *Puma* expression following radiation. Here, wild-type mice were exposed to 7 Gy TBI and collected 3 or 24 hours later. Interestingly, *Puma* mRNA localized to the uterine epithelium and stroma as early as 3 hours postirradiation (Supplemental Figure 5A) and remained abundant at 24 hours (Figure 6A). In accordance, uterine *Puma* mRNA expression was significantly elevated at 24 hours postirradiation when compared with nonirradiated controls (Figure 6B).

Having established that *Puma* expression is induced following radiotherapy treatment, we next exposed *Puma^–/–^* mice to 7 Gy TBI and collected uteri 24 hours later to examine whether loss of PUMA protects the uterus from immediate radiotherapy-induced damage. While irradiated wild-type uteri exhibited DNA damage and apoptosis, as evidenced by the presence of numerous γH2AX- and TUNEL-positive cells, respectively, irradiated *Puma^–/–^* uteri exhibited DNA damage but no apoptosis (Figure 6C).

To investigate whether loss of PUMA could protect uterine function from persistent radiotherapy-mediated damage, artificial decidualization was induced in *Puma^–/–^* mice following TBI. For this experiment, *Puma^+/–^* littermates (i.e., animals with 1 functional copy of the *Puma* gene) were used as controls, and in line with findings in wild-type mice (Figure 4), TBI significantly impaired decidualization (Supplemental Figure 5B). Remarkably, complete loss of PUMA successfully restored the decidual response in irradiated mice, with no significant difference observed in the extent of decidualization when compared to nonirradiated *Puma^–/–^* mice (Figure 6, D and E). Furthermore, when wire myography was performed, TBI induced uterine vasoconstrictive vascular dysfunction in *Puma^+/–^* animals, but uterine artery function was restored in *Puma^–/–^* mice (Figure 6, F–K). Ultimately, embryo transfer experiments demonstrated that pregnancy loss following TBI could be rescued in *Puma^–/–^* mice (Figure 6, L and M). Collectively, these data demonstrate that PUMA-mediated apoptosis is a substantial contributing mechanism of radiotherapy-induced uterine damage and contributor to pregnancy loss observed long-term following TBI.

## Discussion

Using in vivo mouse and in vitro human models, we show that radiotherapy exposure directly damages the uterus and impairs subsequent pregnancy success. Our data demonstrate that (i) previous radiotherapy exposure causes pregnancy loss when healthy, unexposed blastocysts are transferred into irradiated recipient mice; (ii) this pregnancy loss is driven by reduced endometrial receptivity to the later stages of embryo implantation, associated with direct DNA damage and PUMA-mediated apoptosis of uterine cells resulting in impaired decidualization, and reduced uterine blood vessel area and uterine artery endothelial dysfunction; and [iii] genetic loss of PUMA completely restores the decidualization response, uterine artery function, and ongoing pregnancy following radiation exposure.

Using clinically relevant doses of γ-irradiation, pregnancy loss was observed across 3 different radiation doses and conditions. In mice, doses of 9–11 Gy are lethal without intervention, due to depletion of hematopoietic cells (59, 60). Thus, the doses used in the current study of 4.5 Gy and 7 Gy (cumulative) were tolerable. Importantly, pregnancy loss was still observed during abdominopelvic-only exposure when the brain and HPG axis were protected from radiation exposure using lead shielding. This indicates that pregnancy loss is attributable to direct radiation-induced damage to the uterus and not likely to be caused by reduced hormone production due to HPG axis dysfunction. A limitation with the timing of this study comes from the 2-week break between radiation exposure and ovariectomy. While it cannot be ruled out that irradiated animals may have lost ovarian function immediately after radiation and thus, had 2 fewer weeks of ovarian function compared to control animals, critically, all mice received the same exogenous hormone regimen to prepare for and maintain pregnancy. In the future, chemical sterilizing agents that specifically target the ovary would be of great interest to negate the need for ovariectomy at young ages.

It is well established that exposure to γ-irradiation can inflict DNA damage and subsequent apoptosis in a range of cell types (61–63). However, the present study provides what we believe is the first evidence of DNA damage in the mouse uterus and in human endometrial cells following radiotherapy exposure. Moreover, we demonstrate that this damage leads to PUMA-mediated apoptosis of uterine cells. Critically, by utilizing OVX mice to control for irradiation-induced impairment of ovarian endocrine function, we show — for the first time to our knowledge — that this radiotherapy-induced uterine damage occurs directly, independent of ovarian factors. Furthermore, our in vivo mouse models illustrate that this damage causes lasting impacts on uterine function that impair pregnancy establishment and maintenance.

Reports in the literature highlight that women exposed to uterine radiation experience substantially elevated prevalence of complications, including uterine rupture, adhesions, and abnormal placentation (34, 39, 45, 64–67), though the underlying causes and mechanisms have not been clear. As the pregnancy milestones assessed in this study were induced 4 weeks postirradiation (equivalent to approximately 12 months in humans), our data provide what may be the first compelling evidence that the uterus sustains direct damage that persists long-term following radiotherapy. Promisingly, when the same model was applied to apoptosis-resistant *Puma^–/–^* mice, the decidualization response was restored and ongoing pregnancy protected from radiation-induced damage. Indeed, it has been demonstrated previously that *Puma^–/–^* mice deliver healthy litters after exposure to γ-irradiation doses up to 4.5 Gy, while their wild-type counterparts produce no litters (58). However, this study is the first to our knowledge to demonstrate *Puma^–/–^* mice can establish pregnancy following 7 Gy γ-irradiation and distinguishes between the protective effect of PUMA loss in oocytes and embryos, versus the maternal uterine environment following TBI.

Doppler ultrasound is routinely used during pregnancy to monitor fetal growth and development and is useful to assess whether the pregnancy will develop complications (68–70). It is a noninvasive technique from which the pulsatility and resistance indexes of the main uterine artery supplying the uterus can be calculated, indicating the degree of vascular function. In the present study, differences in uterine artery pulsatility and resistance indices were unable to be detected via Doppler ultrasound, potentially due to pregnancy loss already being underway at the time of measurement. However, further investigation of uterine artery function in nonpregnant mice revealed long-term endothelial dysfunction following radiation exposure. Uterine blood vessel remodeling in early pregnancy is critical for healthy pregnancy, with defects in this process known to contribute to the pathology of pregnancy complications such as preeclampsia (71) and fetal growth restriction (72). Investigations in early implantation sites from irradiated animals demonstrated significantly reduced CD31-positive vessel area relative to implantation site cross-sectional area. Thus, our data suggest that radiation-induced uterine artery endothelial dysfunction and impaired blood vessel remodeling in early pregnancy likely contribute to the pregnancy loss observed in our mouse model. Existing literature demonstrates irradiation exposure damages vasculature in other bodily tissues and organs, including the brain (61, 73). Studies in rodents specifically have shown acute irradiation-induced damage to vascular tone that can be restored to unexposed levels by 6 months postirradiation (74, 75). However, the longer term consequences to arise from short-term deficits in blood supply should not be underestimated, as long-term impacts on the function of the affected tissue can arise from hypoxic injury (76, 77). It is likely that downstream damage also occurs to the uterine microvasculature, and this could contribute to both the reduced uterine cellularity in nonpregnant mice and the fetal loss in pregnant mice. Several studies demonstrate that extensive local changes occur in the uterine microvasculature that are essential for successful implantation and placental development (78, 79). The observation of avascular implantation sites in uteri at 3 days after embryo transfer, quantified by reduced CD31-positive vessel area, is consistent with failure of the uterine vasculature to undergo sufficient adaptation for pregnancy.

To elucidate the precise mechanisms of irradiation damage to the uterus, decidualization was artificially induced. Decidualization involves the differentiation of endometrial stromal cells into decidual cells, an event that precedes and underpins healthy placental development. Recent studies have demonstrated defects in decidualization and subsequent placentation are a major cause of embryonic lethality in mouse mutant lines (80), and a contributing factor in age-related reproductive decline (81) as well as gestational disorders including preeclampsia and fetal growth restriction (82, 83). Here, without the presence of a blastocyst, the extent of the decidualization response was impaired in mice exposed to γ-irradiation. Interestingly, the reduced vessel area observed in early implantation sites was concentrated to the decidua, suggesting impaired vessel remodeling is responsible for impaired decidualization. To investigate this, studies were performed in primary human endometrial stromal cells decidualized in vitro, suggesting that the decidualization defect is at least partly intrinsic to radiation exposed stromal cells. Thus, the decidualization process may be hampered during pregnancy establishment in women previously exposed to irradiation because of impaired stromal cell ability to decidualize and reduced vessel remodeling in early pregnancy.

One gap in our study is whether damage to endometrial progenitor cell populations contributes to radiation-induced uterine damage. While reliable markers of human endometrial progenitor cells exist (84, 85), this knowledge is yet to be transferred to murine models (86), with no markers of mouse endometrial progenitor cells currently available. In women, current dogma suggests that endometrial progenitor cells reside in the basalis. The primary human endometrial epithelial and stromal cells utilized in this study were isolated and cultured from Pipelle biopsies, which can be collected only from the functionalis layer. As such, to date, isolation of human endometrial progenitor cells has been possible only through hysterectomy samples, from which potential progenitor populations have been identified based on their ability to form colony-forming units in vitro and flow cytometry sorting (84, 85, 87). Even so, a major limitation in the use of these materials is the very small number of progenitor cells that can be identified, and the difficulty in obtaining sufficient samples. A recent study identified highly proliferative mesenchymal cells, termed decidual precursor cells, from secretory phase endometrial biopsies (88), suggesting that it may be possible to study these progenitor populations using routinely collected Pipelle biopsies in future.

Taken together, this study advances understanding of the specific effects of radiotherapy exposure on the uterus and the underlying mechanisms of this damage. This insight will increase awareness of the uterus as a target for radiation damage and is a step toward future development of effective fertility preservation strategies. The main method for preserving future fertility of women being treated for cancer is the cryopreservation of oocytes and embryos. These options do not prevent ovarian or uterine damage from occurring (89) and confer no protection from pregnancy complications, premature menopause, or loss of endocrine health. Hormone replacement therapy can provide benefits, but it is unable to fully compensate for impaired ovarian hormone production (90). Although GnRH agonists are the current standard of care for prevention of chemotherapy-induced menopause in breast cancer patients, their ability to prevent follicle loss or improve fertility has not been investigated (91). Greater knowledge of how different cancer treatments damage the uterus and ovary is required so that appropriate therapeutic targets can be identified and effective pharmacological options developed.

These findings indicate that protecting the uterus, in addition to the ovaries and oocytes within, is critical to ensure the future fertility, pregnancy success, offspring health, and overall quality of life for female cancer survivors treated with radiotherapy. In particular, we highlight PUMA as a promising therapeutic target and consider that strategies to block PUMA are worthy of investigation, given that previous studies indicate genetic loss of PUMA protects oocytes from both radiotherapy- and chemotherapy-induced damage and preserves long-term fertility in knockout animals (58, 92). Excitingly, a small molecule inhibitor of PUMA has recently become available and has shown promising results in protecting cells from cancer treatment–induced damage (93). However, rigorous preclinical research is required before this can be translated to the clinic. We advocate that fertility preservation techniques aimed at protecting both the ovaries and uterus are necessary to maximize fertility success in female cancer survivors and that studies to advance this goal should be prioritized in the future.

## Methods

### Study design

Until now, the possibility of cancer treatment–induced uterine damage has largely escaped the attention of the fertility preservation field. The goals of this study were to comprehensively determine the extent and mechanisms of uterine damage caused by irradiation exposure and establish whether this affects subsequent fertility and pregnancy success. Carefully designed in vivo models used OVX mice that received exogenous hormones and/or healthy donor embryo transfers in order to study the effects of radiotherapy exposure on the maternal uterine contribution to each early milestone of pregnancy initiation. Additionally, these models circumvented any confounding effects of irradiation exposure on ovarian endocrine function, oocytes, or ensuing fertilization and embryo development. Radiation exposures were chosen based on clinically relevant regimens. Data from these animal models were validated using primary human endometrial cells. Sample size was determined using published work and power calculations. Experimenters were not blinded to treatment groups during the acquisition of data.

### Animals and treatments

All animals were housed in temperature-controlled high-barrier facilities (Monash University Animal Research Laboratory and Experimental Animal Facility), with free food and water access and a 12-hour light/12-hour dark cycle. All procedures were approved by the Monash Animal Research Platform animal ethics committee (Monash University) and performed in accordance with the National Health and Medical Research Council Australian Code of Practice for the Care and Use of Animals.

The goal of this study was to comprehensively determine the extent and mechanisms of uterine damage following irradiation. Adolescent (4-week-old) female mice were used for this study as either untreated control animals or irradiation exposed. All subsequent procedures (ovariectomy, milestones of pregnancy) were completed on animals of all treatment types.

#### Irradiation.

Generation and genotyping of *Puma^–/–^* on a C57BL/6J background have been described previously (94). Four-week-old female C57BL6JMARP/CBAJMARP (F1) or C57BL6JMARP/BALBcJAsmuMARP (F1) or *Puma^–/–^* on C57BL/6J background and their heterozygous littermates (58), were exposed to 4.5 Gy or 7 Gy TBI. Irradiation was delivered as 2 fractions of 3.5 Gy separated by 4 hours, using a double-encapsulated stainless-steel capsule radioactive source containing cesium 137 in the form of cesium chloride. For the lead shielding experiment, mice were anesthetized with ketamine (100 μg/g) and xylazine (10 μg/g), then exposed to 7 Gy as a single fraction. Mice were ovariectomized 2 weeks after irradiation, and another 2-week waiting period was used to enable endogenous ovarian hormone levels to subside before beginning any further experimental procedures outlined below.

#### Artificial endometrial receptivity.

OVX mice were subcutaneously (s.c.) primed with 17β-estradiol (100 ng, MilliporeSigma E8875) on days 1 and 2 and progesterone (1 mg, MilliporeSigma P0130) on days 5–8. Uteri were collected 16 hours after the final injection.

#### Embryo transfer donors.

As previously published (51), adult female BALB/c mice were superovulated using pregnant mare serum gonadotropin (Folligon 5 IU), followed by human chorionic gonadotropin (Chorulon 5 IU), then mated with a proven male BALB/c stud 2 days later. Females were culled and blastocysts flushed from both uterine horns and oviducts 2 days after vaginal plug detection. Blastocysts were cultured overnight in M2 media and transferred to recipient females the following day, with 4–5 blastocysts transferred per uterine horn of each recipient via intrauterine injection.

#### Embryo transfer recipients.

As previously published (51), OVX mice were s.c. primed with 17β-estradiol (100 ng, MilliporeSigma E8875) on day 1, and progesterone (2 mg, MilliporeSigma P0130) on day 3, before embryo transfer on day 4. Four or five donor blastocysts were injected into each uterine horn, for a total of 8–10 blastocysts per animal. Progesterone was supplemented daily until collection at either 3, 7, or 10 days posttransfer.

#### Artificial decidualization.

OVX mice were s.c. primed with 17β-estradiol (100 ng) on days 1–3, then given a s.c. progesterone pellet on day 7 and further 17β-estradiol (5 ng) on days 7–9. Progesterone pellets were made using an established protocol (53, 95). Briefly, silastic tubing was cut into 1 cm–long pieces, sealed at one end with multipurpose sealant and left to cure overnight. Progesterone was added to pellets and the other end sealed. Prior to use, pellets were incubated at 37°C in 1% charcoal-stripped FCS in PBS for 72 hours. Artificial decidualization was induced on day 9, 2 hours after the final hormone injection. A nonsurgical embryo transfer (ParaTechs 60010) device was used to inject 20 μL sesame oil into 1 uterine horn through the cervix. Mice were humanely culled 4 days later, with body and uterine weights recorded to assess the extent of decidualization.

### Histological analysis

Collected tissues were fixed in 10% neutral buffered formalin for 24 hours, embedded in paraffin, sectioned (4–5 μm), and mounted on Superfrost glass slides. Prior to staining, sections were dewaxed in histolene (5 minutes each) and rehydrated through graded ethanols (100%–70%, 2 minutes each) and distilled water (dH_2_O). For counterstaining, slides were placed in Harris hematoxylin (MilliporeSigma HHS16) for 5–15 minutes at room temperature, rinsed with tap water until water was clear, dipped in acid alcohol, rinsed, placed in lithium carbonate for 30 seconds, rinsed, stained with eosin (Amber Scientific; for H&E only) for 2 minutes, and rinsed again. Sections were rapidly dehydrated through graded ethanols (70%–100%), cleared with histolene, and mounted with DPX (Merck HX98094579). Bright-field imaging was completed using a DotSlide microscope (Olympus).

### Immunohistochemistry and immunofluorescence

Formalin-fixed, paraffin-embedded sections (4–5 μm) underwent antigen retrieval using sodium citrate buffer (pH 6.0) or EDTA (Ki67 only). For immunohistochemistry, endogenous peroxidase activity was blocked using 2% H_2_O_2_/PBS for 30 minutes at room temperature. Blocking was performed using 10% serum in TN buffer (0.1 M Tris, 150 mM NaCl) for 30 minutes at room temperature. Primary antibodies (Supplemental Table 2) were incubated overnight at 4°C. After washing, secondary antibody was added for 1 hour at room temperature (Supplemental Table 2). Avidin-biotin complex (VectaStain PK-6100) was added to immunohistochemistry slides for 30 minutes, protected from light at room temperature, followed by DAB (Dako K3468) and a series of washes. For immunofluorescence, slides were mounted with FluorSave reagent (Calbiochem 345789) and imaged using a C1 Inverted Confocal microscope (Nikon). For immunohistochemistry, slides were counterstained with hematoxylin and mounted with DPX (Merck HX98094579), then imaged using a DotSlide microscope (Olympus) or Aperio Digital Pathology Slide Scanner (Leica Biosystems). For CD31 quantification, whole tissue images (3 sections per implantation site, 4 animals per group) were captured with ×20 objective, then visualized and quantified with a blinded assessment protocol using Aperio ImageScope software (Leica Biosystems). The cumulative vessel area per tissue section was measured by performing manual annotations around each vessel. The area of each of the 3 tissues per animal was then averaged to obtain the final representative value of each animal (μm^2^). The vessel area per total tissue area was obtained by dividing the average tissue section vessel area by the total tissue area (μm^2^).

### TUNEL

TUNEL staining was performed using the Chemicon ApopTag kit (MilliporeSigma S7100). Sections were incubated in proteinase K (1 mg/mL diluted 1:50 in PBS) for 15 minutes at room temperature, then quenched with 3% H_2_O_2_ solution for 5 minutes. Equilibration buffer was applied for at least 10 seconds before TdT enzyme (diluted 1:10 in reaction buffer) was added to sections, with reaction buffer alone used as a negative control, for 1 hour at room temperature. Stop buffer solution was diluted in 200 mL dH_2_O, and sections were incubated for 10 minutes. Sections were washed 3 times in PBS, before anti-digoxigenin conjugate was added for 30 minutes. Slides were counterstained with hematoxylin and mounted with DPX.

### In situ hybridization

In situ hybridization was performed using the RNAscope 2.5HD Brown Assay (ACD, 322371) kit, using probes against mouse *Bmp2* and *Ptgs2*, according to manufacturer’s instruction. Bright-field images were captured using DotSlide (Olympus).

### Uterine artery wire myography

Vascular reactivity was assessed as previously described (96, 97) with the following modifications. Mouse uterine arteries were isolated into ice-cold Krebs physiological solution (PSS) containing 120 mM NaCl, 25 mM NaHCO_3_, 5 mM KCl, 1.2 mM MgSO_4_, 1.2 mM KH_2_PO_4_, 11.1 mM d- glucose, and 2.5 mM CaCl_2_. Briefly, main uterine arteries were carefully cleaned of loose connective and adipose tissue. Arteries about 2 mm in length were then mounted on a 4-channel wire myograph (Danish Myo Technology) using 25 μM diameter tungsten gold–plated wire (Goodfellow). Arteries were allowed to stabilize for 15 minutes at 37°C before a stepwise normalization protocol to mimic wall tension of approximately 60 mmHg. All experiments were performed at 37°C in the presence of 95% O_2_ and 5% CO_2_. Changes in isotonic tension were recorded using Powerlab/LabChart data acquisition system (AD Instruments).

To test tissue viability, all arteries were first exposed to high-potassium PSS (KPSS; K^+^ = 100 mmol/L, iso-osmotic replacement of Na^+^ with K^+^) and then washed out. Subsequently, the integrity of the endothelium was determined by submaximally preconstricting arteries with PE (0.1–3 μmol/L) to 60%–70% of KPSS contraction, then applying the endothelium-dependent vasodilator ACh (10 μmol/L) to induce relaxation. Arteries with more than 90% relaxation were deemed suitable for further analysis.

To assess endothelium-dependent and -independent vasodilator function, uterine arteries were precontracted to a similar level (60%–70% of maximum KPSS contraction) using PE (0.1 to 3 μmol/L), and concentration-response curves to the endothelium-dependent agonist ACh (0.1 nmol/L to 10 μmol/L), and the endothelium-independent agonist SNP (0. 1 nmol/L to 10 μmol/L), were determined.

To examine contraction, arteries were exposed to increasing concentrations of PE (0.1 nmol/L to 10 μmol/L), AngII (0.01 nmol/L to 0.1 μmol/L), the thromboxane A2 mimetic U46619 (0.01 nmol/L to 1 μmol/L) or ET-1 (0.01 nmol/L to 0.1 μmol/L).

All vascular drugs were purchased from MilliporeSigma, except for U46619, which was purchased from Cayman Chemicals.

### Ultrasound imaging

Ultrasound imaging was performed using a Vevo2100 system (VisualSonics). Briefly, at 10 days after embryo transfer, mice were anesthetized by isoflurane inhalation and placed in the supine position on a heated imaging platform. As previously described (98), the uterine artery was identified, and then measurements of peak systolic velocity (PSV) and end diastolic velocity (EDV) were averaged across 3 consecutive cardiac cycles. Resistance index (RI = [PSV – EDV]/PSV) and pulsatility index (PI = [PSV – EDV]/velocity time interval) were calculated.

### Human endometrial stromal cell isolation and culture

Human endometrial biopsies were collected at Monash Medical Centre under appropriate Human Research Ethics Committee (Monash University) approvals (0614C, 03066B). Written and informed consent was obtained from each patient before surgery. All experiments were performed in accordance with the National Health and Medical Research Council guidelines for ethical conduct in human research.

Endometrial biopsies were collected by dilation and curettage from premenopausal cycling women (*n* = 4; age range 24–47 years) in the proliferative (*n* = 2) and secretory (*n* = 2) phases of the menstrual cycle. The women had no hormonal treatment for more than 3 months before tissue collection. Primary human endometrial stromal fibroblasts (HESFs) were isolated from biopsies (*n* = 4) as previously described (54, 55) and cultured in DMEM/F12 (Gibco 11330-032) with 10% charcoal-stripped fetal bovine serum (csFBS) and 1% antibiotic-antimycotic (Gibco).

### Chamber slide immunofluorescence

Primary human endometrial stromal cells, and immortalized endometrial epithelial cells (ECC1) (originally from ATCC CRL-2923) were seeded into 8-well Chamber slides (Nunc LabTekII, 154453) at 5,000 cells per well and treated the following day. Cells were permeabilized using 4% paraformaldehyde in 2% Triton/PBS for 15 minutes, then washed with PBS. Blocking was performed using 1% BSA/PBS for 20 minutes, and then primary antibody (γH2AX, Cell Signaling Technology 9718 1:1,000) was applied for 90 minutes at room temperature. A secondary antibody cocktail (goat anti-rabbit Alexa Fluor 488 [1:800], Phalloidin [F-actin; 1:100], Alexa Fluor 568 [Invitrogen A12380; 1:100], and Hoechst [Invitrogen H3569; 1:5,000]) was added to the cells and incubated for 1 hour. Following PBS washes, chambers were removed from Chamber slides and coverslips applied using FluorSave reagent (Calbiochem 345789). Slides were imaged using a C1 Inverted Confocal microscope (Nikon).

### Human endometrial stromal cell in vitro decidualization

HESFs were grown to confluence in DMEM/F12 supplemented with 10% csFBS and 1% antibiotic/antimycotic (Gibco). At 24 hours after irradiation, HESFs were treated with decidualization media containing DMEM/F12 with 2% csFBS, 1% antibiotic/antimycotic, 10^–6^ M estradiol (MilliporeSigma E2758), and 10^–5^ M medroxyprogesterone acetate (MilliporeSigma M1629) (55). Decidualization media were changed every 48–72 hours for 12–14 days, with conditioned media collected for secreted prolactin analysis on days 2, 9, and 12. Cells were collected for RNA isolation on days 12–14.

### Prolactin ELISA

Prolactin ELISA was completed according to manufacturer’s instructions (R&D Systems DY682 and DY008). Briefly, conditioned media from primary human endometrial stromal cells were collected and spun at 590*g* for 5 minutes at room temperature to pellet cell debris. Prolactin standards, blank, and neat conditioned media were loaded (100 μL) in duplicate. The plate was read at 450 nm on a ClarioStar plate reader (BMG), then analyzed by correcting absorbance values to the blank and performing 4-parameter logistic regression analysis.

### Flow cytometry

Mouse uterine single-cell suspensions were made by digesting tissue using collagenase IV (MilliporeSigma C51380; 4 mg/mL), DNase I (MilliporeSigma DN25; 1 mg/mL), dispase (Gibco 170104; 2 mg/mL), and hyaluronidase (MilliporeSigma H3506; 2 mg/mL) in Dulbecco’s PBS (Thermo Fisher Scientific) for 40 minutes at 37°C with agitation (99). Cell suspension was passed through a 70 μm filter (Falcon 352350) to garner a single-cell suspension. Single-cell spleen suspensions were made by physical breakdown of spleens through 70 μm filters. Red blood cells were lysed with red blood cell lysis buffer (supplied in-house through the Monash University Biomedicine Discovery Institute Media Stores), and cells were pelleted and resuspended. A cocktail of directly conjugated antibodies including CD19, CD4, CD8, CD11b, TCRb, F4/80, and NK1.1 was added to uterine and spleen cell suspensions, with unstained spleen cells used to complete compensation (Supplemental Table 3). Cells were Fc-blocked at room temperature before antibody incubation and live/dead staining (Live/Dead Viability stain, BD Horizon FVS-700). Samples were rinsed, pelleted, and transferred to bullet tubes for analysis using Fortessa X20 (BD Biosciences) and FlowJo software (Tree Star Inc.). Cells were gated by live cells and CD45-positive cells, then separated to T cells; B cells; and CD19-, CD11b-, TCRb-, F4/80-, and NK1.1-positive cell populations (Supplemental Figure 1).

### RNA isolation and qPCR

RNA was isolated from whole uteri tissue or cultured cells using the RNeasy Mini Kit (QIAGEN, 74104) according to manufacturer’s instructions. RNA concentration and purity were assessed using a NanoDrop spectrophotometer (Thermo Fisher Scientific). RNA (100–250 ng) was reverse-transcribed and cDNA made using Superscript III first strand synthesis kit (Invitrogen, 18080051). Real-time qPCR was performed using QuantiNova SYBR Green (QIAGEN, 208052) with oligo primer pairs (MilliporeSigma; Supplemental Tables 4 and 5). Expression levels were normalized to housekeeping genes 18s (mouse) and β-actin or GAPDH (human) and analyzed using comparative cycle threshold (ΔΔCT) method as previously described (100). For each analysis, relative gene expression was calculated relative to the average ΔΔCT value for the control samples.

### Data and materials availability

All data are available in the main text or supplementary materials. A material transfer agreement was in place for access to human endometrial biopsies between Amy Winship and Eva Dimitriadis.

### Statistics

All statistical analyses were performed using GraphPad Prism software (Version 8.0). Prior to statistical analysis, all data were assessed for normality using a Shapiro-Wilk normality test. When comparing 2 groups, normally distributed (i.e., parametric) data were analyzed using either an unpaired 2-tailed *t* test or Welch’s *t* test (in cases where variances were unequal), and non-normally distributed (i.e., nonparametric) data were analyzed using a Mann-Whitney *U* test. For 3 or more groups, 1-way ANOVA with either Tukey’s or Holm-Šídák post hoc test was performed for parametric data or a Kruskal-Wallis test for nonparametric data. For wire myography experiments, sigmoid curves were fitted to agonist-induced concentration response data using nonlinear regression to calculate the sensitivity (pEC_50_) of each agonist. Maximum relaxation (R_max_) to ACh and SNP was measured as a percentage of preconstriction to PE. Maximum constriction (E_max_) to PE, AngII, U46619, and ET-1 was measured as a percentage of contraction to KPSS. Group AUC, pEC_50_, R_max_, and E_max_ values were compared between treatment and control using unpaired 2-tailed *t* tests. Statistical significance was set at *P* < 0.05.

### Study approval

All animal studies were approved by Monash Animal Research Platform animal ethics committee (Monash University) (21908, 17971, 15024) and performed in accordance with the National Health and Medical Research Council Australian Code of Practice for the Care and Use of Animals.

Human endometrial cells were isolated from Pipelle biopsy given with informed consent and collected in accordance with Monash Medical Centre Human Research Ethics Committee approvals (Monash University) (0614C, 03066B).

## Author contributions

ALW and KJH conceived the study. MJG, ALW, and KJH designed experiments. MJG, ALW, SAM, FLC, UCS, LRA, JH, and SG performed experiments. MJG, SAM, UCS, LRA, and ALW analyzed data. EM, WZ, ED, SJHC, JFD, PAWR, ASC, SAR, and CEG contributed materials, technical assistance, or intellectual discussion. MJG, ALW, and KJH wrote the manuscript. All authors reviewed and edited the manuscript.

## Supplementary Material

Supplemental data

## Figures and Tables

**Table 1:**
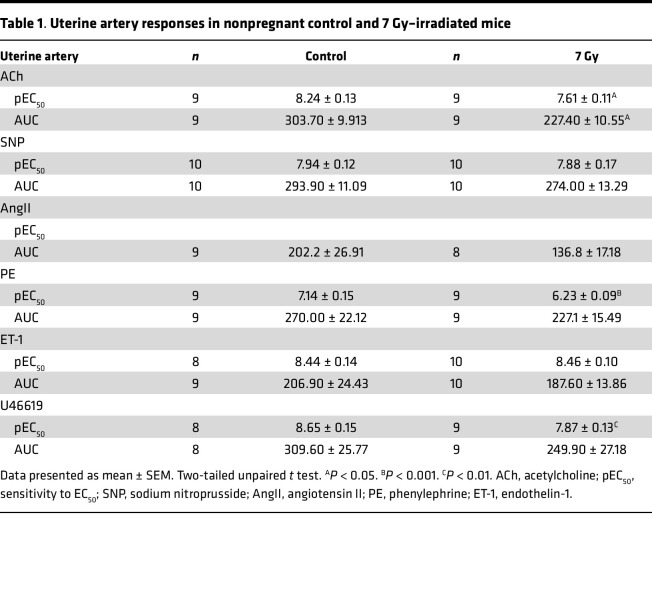
Uterine artery responses in nonpregnant control and 7 Gy–irradiated mice

## Version 1 03/22/2023

Electronic publication
