# Supplementary material for: Radiotherapy exposure directly damages the uterus and causes pregnancy loss
Source: JCI Insight. 2023 Mar 22;8(6):e163704. doi: 10.1172/jci.insight.163704 (PMC10070119; doi:10.1172/jci.insight.163704)
Supplement: Supplemental data [file jciinsight-8-163704-s072.pdf]

Supplementary materials

Radiotherapy exposure directly damages the uterus and causes pregnancy loss

Griffiths et al

SUPPLEMENTARY FIGURE 1

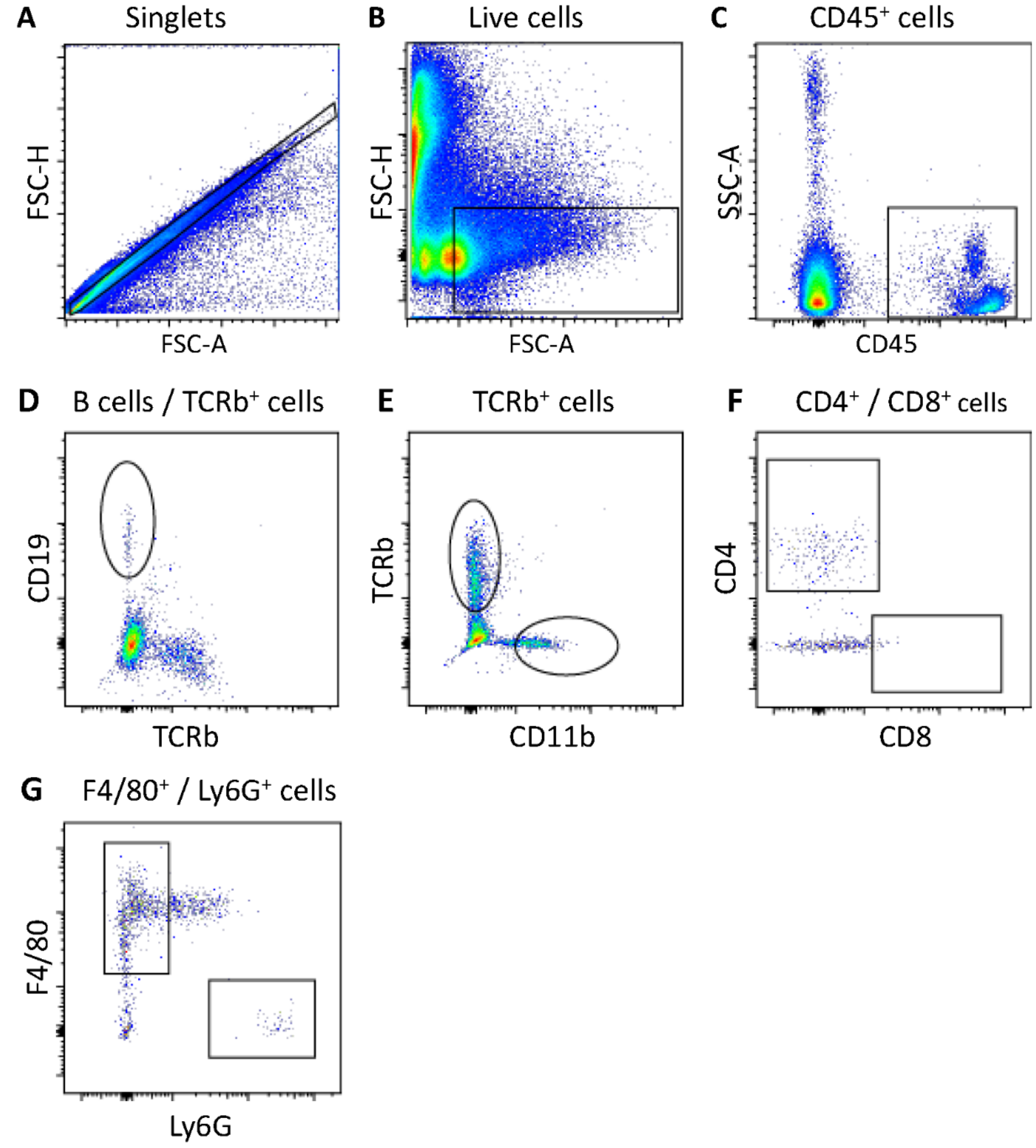

Fig. S1. **Flow cytometry gating strategy.** Cells were identified by gating for singlets (A), and live cells (B). From these, haematopoietic cells were identified by gating for CD45+ cells (C). These cells were separated into CD19+ and TCRb+ cells to identify B and T cells respectively (D), as well as CD11b+ cells by gating against TCRb (E). TCRb+ cells were further defined as CD4+ and CD8+ T cells (F) whereas CD11b+ cells were further defined as F4/80+ and Ly6G+ cells (G).

SUPPLEMENTARY FIGURE 2

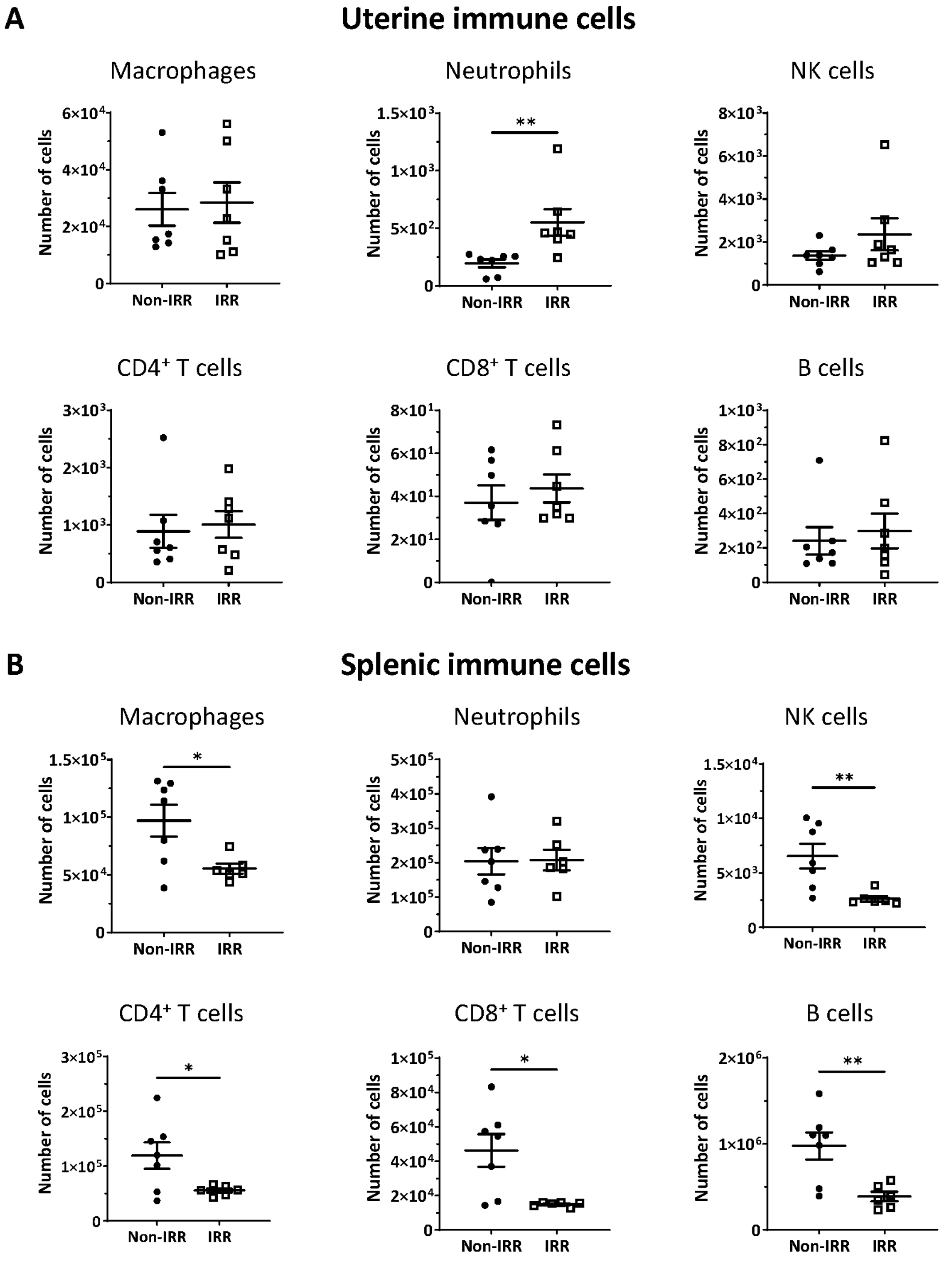

Fig. S2. Uterine cell populations are restored by 4-weeks post-irradiation. Uterine (A) and peripheral (B) immune cell populations were analysed by flow cytometry 4 weeks post-irradiation. Macrophages (i), neutrophils (ii), natural killer (NK) cells (iii), CD4+ T cells (iv), CD8+ T cells (v) and B cells (vi) were quantified. Data are mean ± SEM; unpaired t-test (2 groups; parametric distribution) or Welch's t-test (2 groups; parametric distribution; unequal variances); * = $p<0.05$, ** = $p<0.01$; n=6-7/group.

SUPPLEMENTARY FIGURE 3

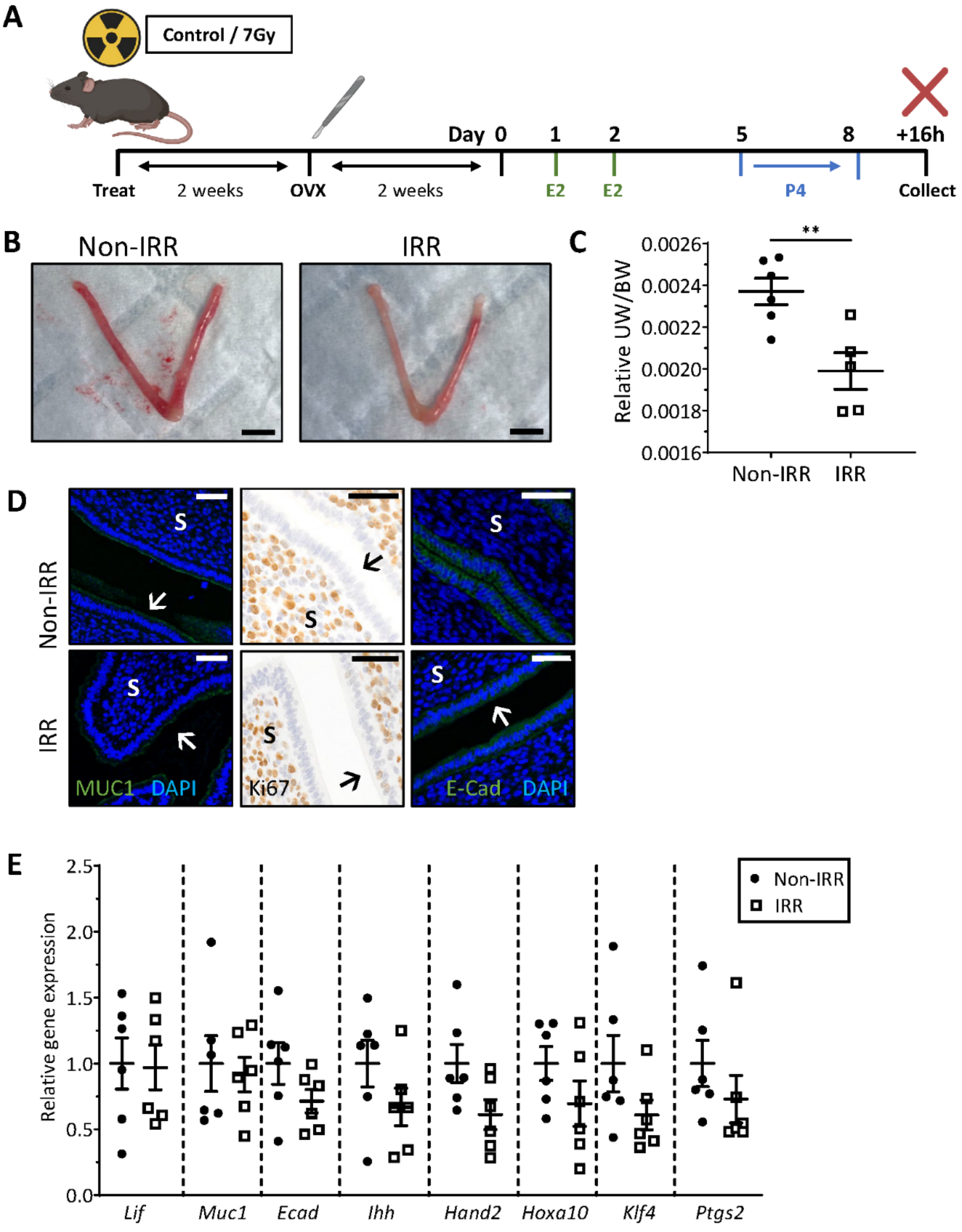

Fig. S3. Endometrial receptivity occurs in female mice following irradiation. (A) Ovariectomised (OVX) adolescent female IRR or Non-IRR wild-type mice were hormone-primed with E2 and P4 to artificially induce endometrial receptivity, then collected 16 h later. (B) Representative images of receptive uteri and (C) relative uteri weight normalised to bod weight. (D) Representative images of immunostaining for MUC1, Ki67 and ECAD (whose expression is lost to attain successful endometrial receptivity) are shown. (E) Expression of genes critical to endometrial receptivity were analysed by RT-qPCR. Data are mean ± SEM; unpaired t-test (2 groups; parametric distribution) or Mann-Whitney test (2 groups; non-parametric distribution); n=5-7/group.

SUPPLEMENTARY FIGURE 4

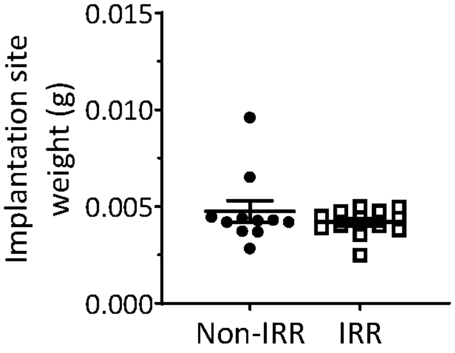

Fig. S4. Implantation site weight unchanged following irradiation. Individual implantation site weights 3-days after embryo transfer are unchanged between non-irradiated and irradiated animals. Data are mean ± SEM; unpaired t-test (2 groups; parametric distribution) or Mann-Whitney test (2 groups; non-parametric distribution); n=11-13/group.

SUPPLEMENTARY FIGURE 5

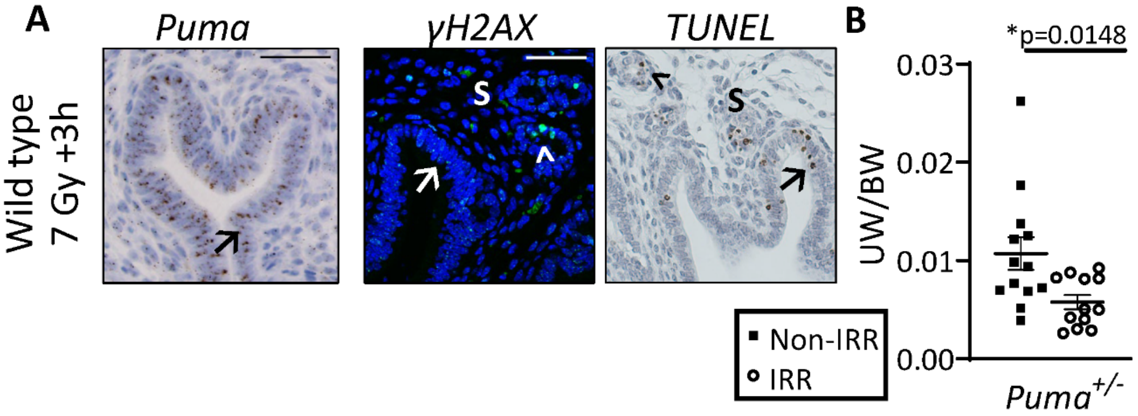

Fig. S5. (A) Activation of *Puma*, DNA damage (γH2AX), and apoptosis (TUNEL) in wild-type mouse uterus 3 hours following irradiaiton**.** (B) $Puma^{+/-}$ mice with one functional copy of *Puma* gene have significantly reduced uterine to body weight ratio following artificial induction of decidualisation. Scale bars are 25μm (*Puma*, γH2AX), or 50μm (TUNEL). → luminal epithelium, S stroma, > glandular epithelium Data are mean ± SEM; unpaired t-test (2 groups; parametric distribution) or Mann-Whitney test (2 groups; non-parametric distribution); n=3-13/group, *p<0.05.

Table S1. Mesenteric artery responses in non-pregnant control and 7Gy irradiated mice. Data presented as mean ± SEM.

Mesenteric artery	**n**	**Control**	**n**	**7Gy**
ACh				
pEC50	7	7.93 ± 0.25	8	7.47 ± 0.45
AUC	8	271.4 ± 32.29	8	213.9 ± 37.63
SNP				
pEC50	7	7.98 ± 0.17	8	8.05 ± 0.19
AUC	8	308.60 ± 29.40	8	285.00 ± 21.22
AngII				
pEC50				
AUC	8	33.49 ± 6.12	7	38.26 ± 11.68
PE				
pEC50	10	6.11 ± 0.12	7	6.13 ± 0.18
AUC	10	192.00 ± 13.77	7	199.2 ± 37.36
ET1				
pEC50	9	9.27 ± 0.36	9	9.34 ± 0.26
AUC	9	289.00 ± 48.43	9	253.20 ± 36.88
U46619				
pEC50	9	8.77 ± 0.23	7	8.67 ± 0.33
AUC	10	396.9 ± 19.62	7	412.20 ± 67.07

Table S2. Immunofluorescence and immunohistochemistry antibodies.

	**Catalogue**	**Species**	**Antigen retrieval**	**Conc**	**Secondary**	**Conc**
**αSMA**	Dako M0815	Mouse	Citrate	1:200	Goat anti-mouse (488)	1:500
**CD31**	R&D Systems AF3628	Goat	Citrate	1:100	Horse anti-goat biotinylated	1:500
**Pan-Cytokeratin**	ab9377	Rabbit	Citrate	1:400	Goat anti-rabbit (568)	1:500
**Cleaved caspase-3**	CST#9661	Rabbit	Citrate	1:100	Goat anti-rabbit (488)	1:500
**E-cadherin**	AF748	Goat	Citrate	1:1000	Donkey anti-goat (488)	1:500
**Desmin**	CST#5332	Rabbit	Citrate	1:100	Goat anti-rabbit (488)	1:500
**Mucin1**	Ab15481	Rabbit	Citrate	1:100	Goat anti-rabbit (488)	1:500
**Ki67**	Ab16667	Rabbit	EDTA	1:500	Goat anti-rabbit biotinylated	1:500
**yH2AX**	CST#9718	Rabbit	Citrate	1:200	Goat anti-rabbit (488)	1:500

Table S3. Flow cytometry antibodies.

	Fluorophore	Laser	Concentration	Company
CD19	BV650	405	1:400	BD Biosciences, 563235
CD4	BUV395	355	1:400	BD Biosciences, 563790
CD25	BV786	405	1:400	BD Biosciences, 564023
CD11b	FITC	488	1:400	BD Biosciences, 557396
TCRb	BV510	405	1:400	BD Biosciences, 563221
F4/80	PE	561	1:200	BD Biosciences, 565410
NK1.1	PE-Cy7	561	1:800	BD Biosciences, 552878
CD8a	PerCP-Cy5.5	488	1:800	BD Biosciences, 551162
CD45	APC Cy7	628	1:400	BD Biosciences, 557659
Ly6G	BV421	405	1:400	BD Biosciences, 562737
Live-dead	FVS700	628	1:2000	BD Biosciences, 564997

Table S4. Mouse primer sequences.

Gene	Forward	Reverse
18s	GTAACCCGTTGAACCCCATT	CCATCCAATCGGTAGTAGCG
Bmp2	GGGACCCGCTGTCTTCTAGT	TCAACTCAAATTCGCTGAGGAC
Ecad	CGTCCATGTGTGTGACTGTG	GCTCTTTGACCACCGTTCTC
Esr1	GCTCCTAACTTGCTCCTGGAC	CAGCAACATGTCAAAGATCTCC
Hand2	TCGGTTATCTAGTGCTGTC	ATACTTACAATGTTTACACCTT
Hoxa10	GCCCCTTCAGAAAACAGTAAA	AGGTGGACGCTACGGCTGAT
Ifng	CGGCTGACCTAGAGAAGACAC	CCAAGATGCAGTGTGTAGCG
Ihh	CTCTTGCCTACAAGCAGTTCA	CCGTGTTCTCCTCGTCCTT
Il10	GCTCTTACTGACTGGCATGAG	CGCAGCTCTAGGAGCATGTG
Il15	CATCCATCTCGTGCTACTTGTGTT	CATCTATCCAGTTGGCCTCTGTTT
Il17	AAGGCAGCAGCGATCATCC	GGAACGGTTGAGGTAGTCTGAG
Il1a	GTATGCCTACTCGTCGGGAG	GGCAACTCCTTCAGCAACAC
Il1b	GAAGAAGAGCCCATCCTCTG	GGAGCCTGTAGTGCAGTTGT
Il23	CCAGCAGCTCTCTCGGAATC	CAGACCTTGGCGGATCCTTT
Klf4	GTGCCCCGACTAACCGTTG	GTCGTTGAACTCCTCGGTCT
Lif	AAAAGCTATGTGCGCCTAACA	GTATGCGACCATCCGATACAG
Muc1	GGCATTCGGGCTCCTTTCTT	TGGAGTGGTAGTCGATGCTAAG
Pgr	CTCCGGGACCGAACAGAGT	ACAACAACCCTTTGGTAGCAG
Ptgs2	AGCCAGGCAGCAAATCCTT	CAGTCCGGGTACAGTCACAC
Tnfa	CCTGGCCTCTCTACCTTGTTG	AGCCTGGTCACCAAATCAGC

Table S5. Human primer sequences.

Gene	Forward	Reverse
PRL	AAAGGATCGCCATGGAAAG	GCACAGGAGCAGGTTTGAC
IGFBP1	AATGGATTTTATCACAGCAGACAG	AATGGATTTTATCACAGCAGACAG
b-ACTIN	TCACCCACACTGTGCCCATCTACGA	CAGCGGAACCGCTCATTGCCAATGG
GAPDH	GAAGGTGAAGGTCGGAGTCAAC	CAGAGTTAAAAGCAGCCCTGGT
